# New Environments Set the Stage for Changing Tastes in Mates

**DOI:** 10.1371/journal.pbio.0030389

**Published:** 2005-10-25

## Abstract

Experimentally manipulating the resource environment of *Drosophila serrata* reveals that mating preferences can evolve, at least in part, as a result of environmentally-based divergent natural selection.

In the evolutionary war of the sexes, females choose their mates while males fight for the right to inseminate. Darwin explained this widely observed phenomenon in terms of energy expenditure: whichever sex invests more to produce and rear offspring gets to choose. That lot typically falls to females, whose mating preferences have driven the evolution of secondary sex characteristics as diverse as the peacock's extravagant tail and the fiddler crab's outsized claw. Such preferences may also influence speciation by causing reproductive isolation, acting as a behavioral barrier to gene flow between populations in much the same way mountain ranges act as physical barriers. In both cases, isolated populations that once interbred can diverge into separate species.

The effect of sexual selection on speciation has been demonstrated in many different organisms, but it's not so clear which evolutionary mechanisms—genetic drift or natural selection—account for the initial shift in mating preferences that generate divergent sexual selection. Different mating preferences could arise as a by-product of chance events related to unique mutations (genetic drift) that produce arbitrary traits later modified by sexual selection, or as a side effect of changes in traits that arise as populations adapt differently to their local environments (divergent natural selection). If divergent selection affects female mating preferences (assuming that mating preference differences contribute to reproductive isolation), then separate populations that adapt to different environments should also diverge in mating preferences, while populations adapted to similar environments should not. [Fig pbio-0030389-g001]

**Figure pbio-0030389-g001:**
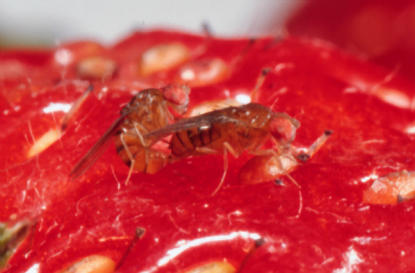
An evolutionary experiment that manipulated the resource environment of Australian fruitflies (shown mating on a strawberry) showed that female mating preference can evolve, at least in part, in correlation with the environment. (Image: A. O'Toole, University of Queensland)

Working with the Australian fruit fly Drosophila serrata, Howard Rundle, Mark Blows, and their colleagues at the University of Queensland in Australia investigated the role of divergent selection in the evolution of female mating preferences. Mate choice in D. serrata is mediated by nonvolatile pheromones in the insect's outer cuticle, called cuticular hydrocarbons (CHCs). In past experiments, male CHCs had been shown to evolve rapidly in response to changes in selection (which is not surprising since they protect the fly against environmental vagaries), but the consequence for female mating preferences was not known.

To address the effect of divergent selection on the evolution of female mating preferences, the authors created different environments in the lab by raising four duplicate fly populations on three different food resources—with yeast representing the ancestral lab environment (these flies have eaten yeast since the stock was established in 1998), and rice and corn representing two novel environments. Flies were raised for 22 months, then fed yeast for two generations to control for environmental effects, before both CHCs and female mating preferences were estimated for each of the 12 populations.

To estimate female mating preferences, a single female from one of the experimental populations was placed in a vial with two males from the ancestral stock population, providing standard males for comparison of preferences among the populations. An average of 106 trials were conducted for each of the populations. After females had mated with one of the two males, CHCs from the chosen and rejected males were extracted for analysis. Female mating preferences were then determined for each population by calculating sexual selection gradients that related the mating success of the males with their CHCs.

CHC profiles for all the flies revealed that nearly every CHC molecule had adapted to the novel environments, although CHC evolution was greater in females than in males. Surprisingly, however, the mating trials showed that female mating preferences had also diverged consistently among populations in correlation with their environment (preferences were similar among populations from the same environment, but differed among populations from different environments). This so-called parallel evolution, the authors argue, implicates divergent selection over drift in preference evolution because genetic drift is unlikely to produce a pattern of preference evolution that is predictable by environment.

Altogether, the authors conclude, this evolutionary experiment shows that mating preferences “can evolve at least in part in correlation with the environment.” This result is consistent with the classic by-product model of speciation, in which new species arise as a side effect of divergent selection; in this case, mating preferences act as a premating isolation mechanism that arises along with the divergent environments. Interestingly, the authors found no correlation between the CHCs that adapted most and those for which female preferences changed. Teasing apart the relative contributions of natural and sexual selection in the evolution of CHCs and mating preferences may help shed light on the complicated relationship between trait and preference evolution in general—and on the role that preference plays in the emergence of new species. —*Liza Gross*

